# Untreated PKU patients without intellectual disability: *SHANK* gene family as a candidate modifier

**DOI:** 10.1016/j.ymgmr.2021.100822

**Published:** 2021-11-19

**Authors:** K. Klaassen, M. Djordjevic, A. Skakic, B. Kecman, R. Drmanac, S. Pavlovic, M. Stojiljkovic

**Affiliations:** a Institute of Molecular Genetics and Genetic Engineering, University of Belgrade, Belgrade, Serbia; b Mother and Child Health Care Institute of Serbia “Dr Vukan Cupic”, Belgrade, Serbia; c School of Medicine, University of Belgrade, Belgrade, Serbia; d Complete Genomics Incorporated, San Jose, California 95134, USA; e MGI, BGI-Shenzhen, Shenzhen 518083, China; f BGI-Shenzhen, Shenzhen 518083, China

**Keywords:** Phenylketonuria, Late-diagnosed, Untreated, Intellectual disability, Modifier gene, SHANK, PKU, phenylketonuria, cPKU, classical phenylketonuria, PAH, phenylalanine hydroxylase, IQ, intelligence quotient, NMDA, N-methyl D-aspartate, NGS, next generation sequencing, ASD, autism spectrum disorders

## Abstract

Phenylketonuria (PKU) is an inborn error of metabolism caused by variants in the phenylalanine hydroxylase (*PAH*) gene and it is characterized by excessively high levels of phenylalanine in body fluids. PKU is a paradigm for a genetic disease that can be treated and majority of developed countries have a population-based newborn screening. Thus, the combination of early diagnosis and immediate initiation of treatment has resulted in normal intelligence for treated PKU patients. Although PKU is a monogenic disease, decades of research and clinical practice have shown that the correlation between the genotype and corresponding phenotype is not simple at all. Attempts have been made to discover modifier genes for PKU cognitive phenotype but without any success so far.

We conducted whole genome sequencing of 4 subjects from unrelated non-consanguineous families who presented with pathogenic mutations in the *PAH* gene, high blood phenylalanine concentrations and near-normal cognitive development despite no treatment. We used cross sample analysis to select genes common for more than one patient. Thus, the *SHANK* gene family emerged as the only relevant gene family with variants detected in 3 of 4 analyzed patients. We detected two novel variants, p.Pro1591Ala in *SHANK1* and p.Asp18Asn in *SHANK2*, as well as *SHANK2*:p.Gly46Ser, *SHANK2*:p.Pro1388_Phe1389insLeuPro and *SHANK3*:p.Pro1716Thr variants that were previously described. Computational analysis indicated that the identified variants do not abolish the function of SHANK proteins. However, changes in posttranslational modifications of SHANK proteins could influence functioning of the glutamatergic synapses, cytoskeleton regulation and contribute to maintaining optimal synaptic density and number of dendritic spines.

Our findings are linking *SHANK* gene family and brain plasticity in PKU for the first time. We hypothesize that variant SHANK proteins maintain optimal synaptic density and number of dendritic spines under high concentrations of phenylalanine and could have protective modifying effect on cognitive development of PKU patients.

## 1 Introduction

Phenylketonuria (PKU; MIM# 261600) is the most common inherited disorder of amino acid metabolism, which is caused by mutations in the phenylalanine hydroxylase (*PAH*) gene [1]. Before newborn screening was introduced, it was reported that 87.6% of untreated PKU patients have IQ lower than 40, and only 0.6% had near-normal or normal cognitive development (IQ >80) [2]. Now, PKU is a metabolic pathology that represents the paradigm of a hereditary disease that can be treated – the implementation of low phenylalanine diet and the use of BH4 (sapropterin dihydrochloride) treatment have enabled PKU patients to evade the development of mental retardation and attain normal cognitive development. It is well documented that the phenylalanine levels during the neonatal period are clearly and negatively related to later IQ [3]. With the majority of the countries worldwide performing neonatal PKU screening, untreated PKU patients are now rare to be found in routine clinical practice. Nevertheless, patients that were born before their national PKU screening began, or patients that were lost to follow-up and/or had a poorly controlled or late introduced diet are still possible to be detected [4].

Even though two disease-causing mutations in the *PAH* gene stand as the main determinant of PKU phenotype severity, genotype–phenotype inconsistencies have also been reported [5], [6]. Therefore, a model of PKU as a ‘simple’ Mendelian disorder should be shifted towards the understanding of a more complex disease phenotype, leading to a search for additional factors needed for a more sophisticated genotype – phenotype correlation [7], [8]. Given that the phenylalanine metabolism (and consequently its blood level) is very much complex itself, it could be presumed that the additional factors contributing to final PKU phenotype represent yet undiscovered modifier genes involved in the regulation of any step of phenylalanine metabolism. However, the majority of inconsistensies have been noted in cognitive PKU phenotype, with cases including siblings with identical genotype and different clinical outcome and even untreated PKU patients that have reached near-normal cognitive development [4], [9], [10]. It has been proposed that protective variants in putative modifier genes could be accounted for these atypical cases. Therefore the search was mainly aimed at the blood brain barrier transport of phenylalanine, along with ascertaining the specific way phenylalanine damages the neurons and the nervous system. A recent study examined cohort of 28 patients with severe PKU that were timely diagnosed and treated since they were 2–6 months old [11]. Patients did not adhere to dietary recommendations prior to the MRS examination in order to allow for the measurement of their brain phenyalalnine levels. This study associated the intronic rs113883650 variant in *LAT1* (*SLC7A5*) gene with increased brain phenylalanine concentration in PKU patients. On the other hand, there are also prevalent viewpoints that it is actually the lack of other large neutral amino acids that impairs protein synthesis in the brain and thus leads to neurodegeneration [12], [13].

Abundant evidence shows that phenylalanine itself has toxic effect on the fragile developing central nervous system [14], [15], [16]. Elevated phenylalanine was shown to impair myelination, whereas white matter disturbances were frequently reported in PKU, especially in untreated patients [17]. Moreover, it has been shown that elevated phenylalanine concentrations in mouse primary cortical neurons lead to reduced synaptic density and alter dendritic branching [18]. Furthermore, studies have identified elevated phenylalanine as the modulator of glutamatergic synapses responsible for learning and memory forming [19]. Still, the exact way by which phenylalanine causes all these alterations is not completely elucidated yet. One of the possible causes is that phenylalanine leads to oxidative stress which in turn causes neurodegenaration [20], but the research continues and the growing evidence points to a complex interplay between phenylalanine, neurons, microglia, deregulated immune response, and quite possibly, genes and neural pathways with yet undiscovered roles.

Identifying individuals resilient to damaging phenylalanine effect might aid to reveal protective variants in genes involved in pathways representing targets of phenylalanine neurotoxic effects. Furthermore, these putative modifier genes could shed new light on the mechanism by which phenylalanine damages the central nervous system and causes cognitive impairment. At the end, discovery of novel candidate PKU modifiers could contribute to better understanding of complex PKU phenotype and have therapeutic implications for the patients. In this study, we have analysed 4 untreated PKU patients without intellectual disability using whole genome sequencing in an attempt to identify candidate PKU modifier genes.

## 2 Subjects and methods

PKU patients were evaluated at the Mother and Child Health Care Institute of Serbia “Dr Vukan Cupic” in Belgrade, which serves as the national referral center for inherited metabolic diseases, hosts PKU newborn screening program (established in 1982 in Serbia) and harbors the only metabolic laboratory in the country. All patients were further referred to the Institute of Molecular Genetics and Genetic Engineering, University of Belgrade, where molecular genetic analysis was performed. Cohort of 61 patients have been previously described [5], [21]. Since 2013, additional 17 PKU patients were diagnosed providing a final cohort of 78 PKU patients. All available parents' samples were routinely analysed to confirm segregation of variants in the family. Although most PKU patients were detected by the newborn screening program, some were diagnosed later in life during evaluation of psychomotor retardation or by routine measurement of blood phenylalanine level at the Mother and Child Healthcare Institute “Dr Vukan Cupic” in Belgrade.

Subjects without intellectual disability who were previously not treated were either first diagnosed after giving birth to a child with PKU (P1 and P2) through the routine segregation analysis of parents, or due to the failure to thrive at the age of 1y 3 m and 1y 4 m (P3 and P4 respectively). These four subjects were from unrelated non-consanguineous families. They were confirmed to have two pathogenic mutations in the *PAH* gene and high blood phenylalanine concentrations. Patients were classified into classical PKU (cPKU) according to pretreatment phenylalanine (Phe) blood level as described in [5]. Cognitive development (evaluated as IQ scale) of the patients was assessed using age-appropriate Witti test, with near-normal cognitive development as defined by an IQ ≥ 80. Genotype and phenotype data of PKU patients with near-normal cognitive development are given in Table 1.

**Table 1:** Genotype and phenotypic characteristics of untreated PKU patients with near-normal cognitive development.

Patient number	Gender	Age at diagnosis	*PAH* genotype	Phenylalanine level (μmol/l)	Metabolic phenotype	Dietary treatment	IQ⁎	Social aspects
P1	F	18y	p.Leu48Ser/p.Leu48Ser	>1200	cPKU	Never applied	80–85	Finished elementary school, lives independently at home
P2	F	21y	p.Leu48Ser/p.Leu48Ser	>1200	cPKU	Never applied	80–85	Finished elementary school, lives independently at home
P3	F	1y 3 m	p.Leu48Ser/p.Leu48Ser	>1200	cPKU	Never applied	85	NA
P4	F	1y 4 m	p.Leu48Ser/p.Arg408Trp	>1200	cPKU	Low phenylalanine diet only between 22 m – 5y	110	University degree, lives independently at home

⁎ IQ was measured at the time of diagnosis, except for P4 (at the age of 35y)

We performed the whole genome analysis of these four “unusual” patients. Additionally, we performed whole genome sequencing of offsprings from mothers with previously undiagnosed PKU. Each mother had one child with PKU who was diagnosed at newborn screening. Both children were born from uncontrolled pregnancies regarding the level of phenylalanine and poorly adhered to the prescribed low phenylalanine diet. There were no blood relatives with late-diagnosed PKU who developed mental retardation.

Moreover, a total of 143 unrelated Serbian individuals (84 males and 59 females), that were previously analyzed by NGS approach using the Illumina Clinical Exome Sequencing TruSight One Gene Panel (Illumina, San Diego, CA, USA), described in [22], were used as the in-house population specific database.

This study has been approved by the Ethics Committee of the Mother and Child Health Care Institute of Serbia „Dr. Vukan Cupic“ in Belgrade, Serbia and has therefore been performed in accordance with the ethical standards laid down in the 1975 Declaration of Helsinki and its later amendments. Informed consent was obtained from all individual participants included in the study and/or their legally authorized representatives.

### 2.1 Genetic and computational analysis

Genomic DNA was isolated from blood using QIAamp DNA-Blood-Mini-Kit (QIAGEN, Hilden, Germany). Whole Genome Sequencing was performed at Complete Genomics, Mountain View, California, USA (http://www.completegenomics.com) [23]. As a reference genome, GRCh37 genome was used.

Bioinformatics analysis started with all genes in which variants were detected using whole genome sequencing, and the following filters were used:

a) Variants that didn't pass variant call quality filters (Q20 > 95%, Q30 > 85%, Depth > 100×/180×, 30× Coverage ≥95%) were excluded.

b) Variants with minor allele frequencies >1% in gnomAD were excluded.

c) Synonymous variants were excluded, while missense, stop-gain, stop-lost, splice site, frameshift and small in-frame indels variants were kept.

d) All ACMG categories of variants (benign, likely benign, variants of uncertain significance, likely pathogenic and pathogenic) were kept.

e) Genes which function is not related to central nervous system functioning were excluded. This was determined using Human Phenotype Ontology (https://hpo.jax.org/app/) and based on information from NCBI (http://www.ncbi.nlm.nih.gov), GeneCards (http://www.genecards.org) and OMIM (https://www.omim.org).

The remaining genes included genes coding for: transporters (especially if located on blood-brain barrier) and enzymes in the brain (*SLC15A5, SLC24A1, SLC35F5, SLC45A4, SLC4A9, SLC6A3, SLC9A5, MFSD6L, ATP8B3, CES1, TH, NPEPS, DBH*), proteins involved in brain development and neuronal differentiation (*PHF2, CDHR2, DOPEY2, AHNAK, EFHD1, DCDC2B*), proteins previously associated with neurodegeneration, neuronal function disorders, neurological disorders and cognitive dysfunction (*ERVW-1, GOLGA6L2, MED25, DNAH14, ANK3, LYPD2, NAPRT1, C10orf112 (MALRD1)*), *C3orf58, C6orf130 (OARD1), VPS13A*), proteins participating in neuronal connections with ion channels and associated proteins (*AHNAK2, ANO10, CACNA1H, CADPS2, CLDN23, CLDN8, CNTNAP2, CTBP2, KCNV2, MARVELD3, NLGN2, OTOF, PCDHA4, PCDHGB6, PCLO, PKP3, PPP1R9B, RIMBP2, RIMS1, SHANK1, SHANK2, SHANK3, TSPAN16*), receptors expressed in brain and neuronal signal transduction proteins (*PTPRT, HTR2C, NRP2*) etc.

Detected variants were checked if previously described in the Human Gene Mutation Database, ClinVar or VarSome databases and further classified acccording to ACMG guidelines. The effect of novel genetic variants was analyzed using different softwares: PolyPhen-2 (http://genetics.bwh.harvard.edu/pph2), SIFT/PROVEAN (http://www.provean.jcvi.org) and MutPred (http://mutpred.mutdb.org). Selected identified variants were confirmed by conventional Sanger sequencing.

### 2.2 *In silico* modelling of SHANK proteins

For *in silico* models of SHANK1 and SHANK3 protein, we used AlphaFold algorithm to predict the 3D structure from amino acid sequence, using the PDB codes AF-Q9Y566-F1 for SHANK1 and AF-Q9BYB0-F1 for SHANK3 [24]. For SHANK2 protein, we used the Phyre2 web portal for prediction of the structure, using the PDB code c5g4xA_ of the SHANK3 N-terminus crystal structure as the template [25]. The images were prepared using the PyMOL Molecular Graphics System, Version 2.4, Schrödinger, LLC.

## 3 Results

In this research, we analysed complete genome sequence of 4 untreated PKU patients who escaped intellectual disability. All patients had two mutations in the *PAH* gene, they were classified to have classic PKU according to metabolic phenotype, but, nevertheless, attained near-normal cognitive development. Therefore, this cohort represents a favorable group for identifying candidate modifiers of PKU cognitive phenotype.

The complete list of variants identified in genes (68 variants in 52 genes) related to central nervous system functioning in the cohort of untreated PKU patients without intellectual disability is provided in the Supplementary Table 1.

Firstly, we searched for the variants in genes that were previously marked as PKU modifiers, namely: *LAT1* (*SLC7A5*) and *4F2hc* (*SLC3A2*). We did not detect any variants in these genes in the patients from our cohort.

Furtheron, the key approach in the analysis was to find what these 4 untreated patients with near-normal cognitive outcome have in common. Therefore, we used cross sample analysis to select genes common for more than one patient. Six variants, 8.8% of all selected variants (in *CDHR2, DCDC2B, NPEPPS, PCDHA4, PPP1R9B, TSPAN16* genes) were found in two unrelated patients (Supplementary Table 1). In five genes (*AHNAK, CDHR2, CLDN23, PTPRT, SLC4A9*), 9.6% of all selected genes, different variants were identified in two unrelated patients. In only one gene (1.9%), namely *VPS13A,* we detected different variants in three unrelated patients. In addition, we identified different variants in three unrelated patients of one gene family (*SHANK1, SHANK2* and *SHANK3*).

*VPS13A* gene is associated with development of a neurological disorder. It encodes a vacuolar sorting protein 13A which is ubiquitously expressed in human tissues, including brain. It is a peripheral membrane protein which is associated with multiple organelles and influences mitochondrial morphology and lipid droplet motility [26] thus not being highly consistent with pathophysiology of PKU.

Therefore, *SHANK* gene family emerged as the only relevant gene family with variants detected in 3 out of 4 analyzed patients. *SHANK* (SH3 and multiple ankyrin repeat domains) family consists of 3 genes (*SHANK1*, *SHANK2* and *SHANK3*). Given that SHANK proteins are involved in synaptic transmission [27], we proceeded with the analysis of detected variants in *SHANK* genes.

We detected one missense variant in *SHANK1* and one in *SHANK3*, while in *SHANK2* we detected two missense variants and one small in-frame insertion (Table 2), with all variants detected in heterozygous state. All variants were confirmed by conventional Sanger sequencing.

**Table 2:** Variants identified in *SHANK* gene family in the cohort of untreated PKU patients without intellectual disability.

Patient	Gene	Genomic coordinates at hg19	Reference sequence	Genetic variant		Variant type	GnomAD frequency	Reference
				Nucleotide change	Amino acid change			
**P2** (cPKU, IQ80-85)	SHANK1	chr19:51170446 G/C	NM_016148.5	c.4771C > G	p.Pro1591Ala	missense	0.00001578	**This study**
	SHANK3	chr22:51169504 C/A	NM_001372044.2	c.5146C > A	p.Pro1716Thr	missense	0.003056	[30]
**P3** (cPKU, IQ85)	SHANK2	chr11:70332231 G/GAATGGC	NM_012309.5	c.4166_4167insGCCATT	p.Pro1388_Phe1389insLeuPro	in-frame insertion	0.001525	[29]
	SHANK2	chr11:70858237 C/T	NM_012309.5	c.136G > A	p.Gly46Ser	missense	0.0003531	[28]
**P4** (cPKU, IQ100)	SHANK2	chr11:70858321 C/T	NM_012309.5	c.52G > A	p.Asp18Asn	missense	0.00002133	**This study**

In P1 we did not detected variants in SHANK gene family. Variant c.4166_4167insGCCATT (p.Pro1388_Phe1389insLeuPro) was previously reported in [29] as c.3024_3029dup, (p.Leu1008_Pro1009dup) at the NM_001379226.1; variant c.5146C > A (p.Pro1716Thr) was previously reported in [30] as c.4960C > A (p.Pro1654Thr) at the NM_033517.1.

**Fig. 1:**
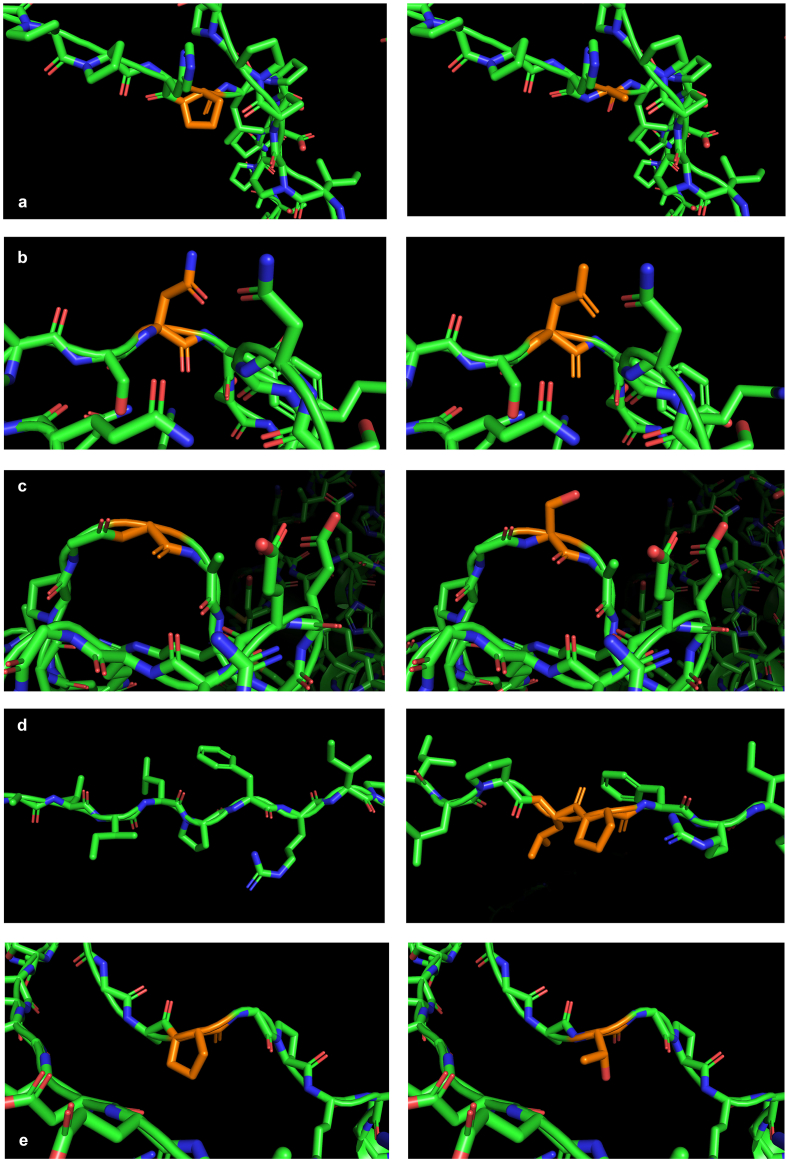
Three-dimensional molecular models of SHANK proteins with close-up view of the regions harboring novel variants. Residues affected by variants are depicted in orange. First inline image represents wt protein whilst the image next to it represents the variant protein. a) SHANK1:p.Pro1591Ala; b) SHANK2:p.Asp18Asn; c) SHANK2:p.Gly46Ser; d) SHANK2:p.Pro1388_Phe1389insLeuPro; e) SHANK3:p.Pro1716Thr. MutPred software additionally explained subtle changes seen in the three-dimensional molecular models: for SHANK1:p.Pro1591Ala - loss of glycosylation at Pro; for SHANK2:p.Asp18Asn loss of stability; for SHANK2:p.Gly46Ser – gain of loop; for SHANK2: p.Pro1388_Phe1389insLeuPro – in-frame insertion of two amino acids; for SHANK3:p.Pro1716Thr – gain of phosporylation at threonine residue.

Out of detected variants, *SHANK2*:p.Gly46Ser, *SHANK2*:p.Pro1388_Phe1389insLeuPro and *SHANK3*:p.Pro1716Thr were previously desribed [28], [29], [30]. All detected variants are considered to be rare, with frequency less than 0.01 in gnomAD (MAF <1%). Furthermore, we checked if variants in *SHANK2* and *SHANK3* were present in our ih-house population specific TruSight One derived database consisted of 143 patients, where only *SHANK2*:p.Gly46Ser was detected in one patient. Variants were additionaly characterized using computational analysis by individual algorithms such as PolyPhen-2, SIFT/PROVEAN and MutPred, where all variants were regarded as benign except for *SHANK2*:p.Asp18Asn which was considered as neutral/probably damaging. Therefore, computational analysis indicated that the identified variants do not abolish the function of SHANK proteins. We performed *in silico* modelling of SHANK proteins to show the effects of all the variants upon the structure (Fig. 1). For SHANK1:p.Pro1591Ala we found loss of glycosylation at proline; for SHANK2:p.Asp18Asn loss of stability; for SHANK2:p.Gly46Ser – gain of loop; for SHANK2: p.Pro1388_Phe1389insLeuPro – in-frame insertion of two amino acids; for SHANK3:p.Pro1716Thr – gain of phosporylation at threonine residue. Neither of these changes are damaging. However, changes of posttranslational modifications at proline residues which are located in the proline-rich domain of SHANK proteins could influence functioning of the glutamatergic synapses, cytoskeleton regulation and contribute to maintaining optimal synaptic density and number of dendritic spines.

## 4 Discussion

Recent reports of individuals with disease-causing mutations in the *PAH* gene and near normal cognitive development are emerging [4], [31] as these patients represent a very interesting group in terms of elucidation of different mechanisms involved in PKU pathophysiology.

In this study, we presented 4 individuals, each individual harboring two pathogenic mutations in the *PAH* gene and a clinical phenotype resilient to mental retardation. Both mutations, p.L48S and p.R408W are well-known fully penetrant mutations, and *PAH* genotypes detected in these individuals are the most frequent p.[Leu48Ser];[Leu48Ser] and the second most frequent p.[Leu48Ser];[Arg408Trp] in Serbian PKU patients [32]. Some of these individuals were born before PKU newborn screening was introduced in Serbia in 1982. However, even in countries where a systematic neonatal screening for PKU has been implemented for 40 years, there are still important risks in finding patients with undiagnosed PKU, in particular those having a PKU child [33]. It was calculated that the minimal risk for a PKU child to have one of his parents with PKU in Serbia is about 1/83. In fact, we have detected two PKU mothers in the cohort of 78 PKU patients.

Patients analysed in this study were classified to have classic PKU according to their metabolic phenotype, but unexpectedly, they attained near-normal cognitive development despite no treatment. Application of NGS technology in this type of samples has major advantages since it enables the identification of novel modifier genes. Since the hallmark of untreated PKU is mental retardation, and that our cohort escaped intellectual disability, we focused on the genes coding for proteins involved in central nervous system functioning.

Firstly, we searched for the variants in genes that were previously marked as PKU modifiers. Interestingly, we did not detect any variants in two genes coding for the heterodimeric transporter, *LAT1* (*SLC7A5*) and *4F2hc* (*SLC3A2*), for which earlier studies showed that they could be potential modifiers of PKU phenotype [34], neither did we detect the intronic rs113883650 variant in the *LAT1* (*SLC7A5*) gene which has been associated with increased concentration of brain phenylalanine in PKU patients with severe hyperphenylalaninemia as shown in a recent study [11].

In order to increase the power of our study, we searched what these 4 untreated patients with near-normal cognitive outcome have in common. Therefore, we used cross sample analysis to select genes common for more than one patient. Although all four subjects did not have a single variant or even variants in single genes in common, three of them had variants in *VPS13A* gene and in three genes of the same family, comprising of *SHANK1*, *SHANK2* and *SHANK3* genes. *VPS13A* gene encodes a peripheral membrane protein which is associated with multiple organelles and influences mitochondrial morphology and lipid droplet motility [26], therefore not looking as a strong PKU modifier gene candidate.

Members of SHANK protein family are scaffold proteins that localize to postsynaptic density of dendritic spines in excitatory synapses in the brain [27]. SHANK proteins are involved in the organization of the glutamatergic synapse by linking receptor proteins on the postsynaptic membrane to actin cytoskeleton of the dendritic spine. It has been shown that these proteins indirectly interact with NMDA receptor complex, metabotropic glutamate receptors and adapter proteins associated to actin filaments, so they can be considered key to regular functioning of the glutamatergic synapses [35]; [36]. Furthermore, it has been shown that SHANK proteins also mediate the maturation of neuronal dendritic spines, a process that is extremely important for the plasticity of the nervous system [30]. The availability of several SHANK mutant rodents and the generation of patient-specific induced pluripotent stem cells (iPSC) have been helping to fully clarify the function of SHANK proteins in the correct neuronal and synapses development [37], [38].

Interestingly, the changes in dendritic spines along with the disturbances in the glutamatergic synapse functioning are observed in PKU animal models [18]. Studies on mice and rat primary cortical neurons have shown that elevated phenylalanine reduces synaptic density and number of dendritic spines [18], [39]. Furthermore, several studies have shown glutamatergic synapse dysfunction in elevated phenylalanine conditions [19], [40]. Possible mechanism is explained by the discovery that phenylalanine, in concentrations comparable to the ones measured in brains of cPKU patients, causes significant and selective decrease of NMDA receptor currents, given that it acts as its partial agonist [41], [42]. Considering the important roles of SHANK proteins in functioning of the glutamatergic synapses and maturation of neuronal dendritic spines, as well as their interaction with the NMDA receptor, it could be assumed that these proteins could somehow be involved in the development of final PKU cognitive phenotype. We hypothesized that one of the factors contributing to the inter-individual differences in brain vulnerability to high phenylalanine between PKU patients may be due to variants in *SHANK* gene family. Our findings designate *SHANK* gene family as a candidate modifier, not a definite modifier of PKU.

Variants detected in patients analyzed in this study are missense and one small in-frame insertion, and *in silico* analysis indicated they are not pathogenic, meaning they will not cause diseases related to these genes, such as Phelan-McDermid syndrome, authism or schizophrenia. Variants in *SHANK* genes that lead to neuropsychiatric disorders and cognitive dysfunction are mostly large deletions which abolish the gene function [30]; [43], although point mutations have also been regarded as pathogenic and disease causing, especially if they disrupt the conserved domains of these proteins [29]. While damaging variants in *SHANK* genes are reported to cause neuropsychiatric disorders, benign variants in *SHANK* genes may have different role precisely because they are present in individuals who escaped intellectual disability. This is not the first time that the NGS methodology pointed to a benign variants as protective (benign missense variant in *REST* gene is protective for hippocampal atrophy) [44].

*SHANK2*:p.Gly46Ser was noted in a large GWAS study by Richards et al. in an effort to explore rare variants contributing to schizophrenia, but it was not genome-wide significant. This variant was also present in our in-house population specific TruSight One derived database. Variant *SHANK2*:p.Pro1388_Phe1389insLeuPro was initially discovered in a patient with ASD and his unaffected mother by [29] (where it was described as L1008_P1009dup as the exons of the neuronal isoform of *SHANK2* were sequenced), but further analysis of the patient yielded additional rare variants that potentially contribute to the disease, so this variant itself could not be designated as the cause of ASD phenotype. *SHANK3:*p.Pro1716Thr was detected in a patient with ASD, but also in healthy controls (labeled *SHANK3:*Pro1654Thr as the NM_033517.1 was used as a reference sequence) [30]. Therefore, none of the previously desrcibed variants can be regarded as disease causing.

Additionally, we detected two novel variants, SHANK1:p.Pro1591Ala and SHANK2:p.Asp18Asn. Both variants are present in a very low frequency in the GnomAD database (Table 2). Variant *SHANK2*:p.Asp18Asn is located in the first coding exon (exon 3) of *SHANK2* gene. This region of the protein precedes the ankyrin repeat domain which is also involved in protein-protein interaction and actin cytoskeleton regulation [43]. A change from an acidic side chain residue to the one with a carboxamide side chain could therefore subtly impact the protein and perhaps alter its characteristics leading to loss of stability as predicted by MutPred. Patient harboring this variant was born before national neonatal screening program was introduced, so she had a late diagnosis at age two, when PKU diet was introduced, but it was discontinued at age five. Nevertheless, she attained normal cognitive development with an IQ of 110 and she even graduated from college. Her *PAH* genotype comprises the severe p.Arg408Trp mutation in addition to p.Leu48Ser, which resulted in her blood phenylalanineof 1200 μmol/l and a phenotype of cPKU. The sharp inconsistency between her metabolic and cognitive phenotype is therefore strongly suggestive of a protective modifier presence.

**Fig. 2:**
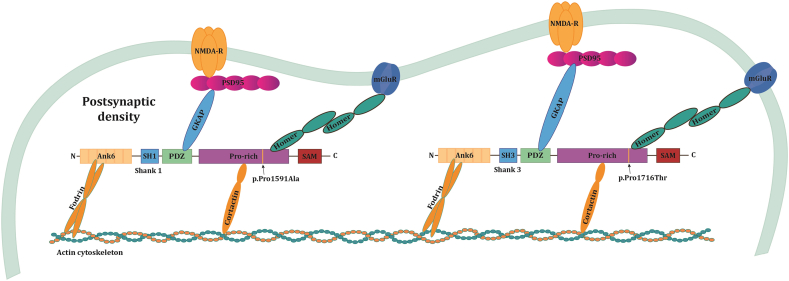
Organization of postsynaptic density with SHANK proteins as central adapters. Variants p.Pro1591Ala and p.Pro1716Thr, found in SHANK1 and SHANK3 respectively, are located in the Proline-rich protein domain which interact with Homer and Cortactin proteins thus enabling regular functioning of the glutamatergic synapses and the cytoskeleton regulation.

Variant p.Pro1591Ala is located in the exon 23 (of 24 exons in total) of *SHANK1* gene and the amino acid change is predicted to lose glycosylation at proline residue. *SHANK1* exon 23 is exceptionally large and encompasses the proline-rich domain (Fig. 2). This domain binds to adapter proteins such as Homer, which binds to metabotropic glutamate receptors, and cortactin proteins, which interact with actin and are therefore involved in cytoskeleton regulation [43]. Patient harboring p.Pro1591Ala variant in SHANK1 (P2), has an additional previously reported p.Pro1716Thr variant in SHANK3 which is predicted to gain phosporylation at threonine residue. Given that both these variants are located in the proline-rich domain, and in both cases the residue affected by the variant is proline, the amino acid change may somewhat affect the structure of the proline-rich domain and the fine tuning of SHANK's interations with metabotropic glutamate receptors and the cytoskeleton regulation.

Interestingly, the son of P2 also carries heterozygous variants p.Pro1591Ala in SHANK1 and p.Pro1716Thr in SHANK3. He was diagnosed through neonatal screening while his mother was diagnosed by chance in adulthood only upon the diagnosis of the son. Therefore, she led a normal life, attained near-normal cognitive development (IQ ranging from 80 to 85), was never suspected of having PKU and was never treated. With blood phenylalanine of 1200 μmol/l she was classified as cPKU. Although her son was born from uncontrolled pregnancy with high blood levels of phenylalanine and poorly adhered to low-phenylalanine diet himself, he also escaped the intellectual disability (IQ 80–85) indicating a possible joint modifying role of p.Pro1591Ala in SHANK1 and p.Pro1716Thr in SHANK3.

Even after decades of studying molecular pathophysiology associated with PAH deficiency, the precise cause of brain dysfunction due to high phenylalanine levels remains incompletely understood [45]. Knowing that the elevated phenylalanine reduces synaptic density and number of dendritic spines [18], [39], [46] and that SHANK proteins are localized in dendritic spines and mediate its maturation during brain development, findings from our cohort of untreated PKU patients with near-normal cognitive development are linking *SHANK* gene family and brain plasticity in PKU for the first time. We hypothesize that variant SHANK proteins maintain optimal synaptic density and number of dendritic spines (through regulation of functioning of glutamatergic synapses and the cytoskeleton) under high concentrations of phenylalanine. Given the small sample size of this study, it should be noted that *SHANK* genes should be screened for in a larger study of untreated PKU patients who managed to escape intellectual disability. Further functional studies, ideally in the cortical neurons differentiated from patient-specific iPSC, are needed to confirm our hypothesis and determine the exact mechanism how identified variants in *SHANK* genes maintain dendritic spines under high phenylalanine concentrations. Furthermore, having in mind the complexity of brain development and brain plasticity, *SHANK* gene family may be one of the first, but certainly not the only player contributing to the inter-individual differences between PKU patients.

Two hypothetical mechanisms that could explain some resilience of brain functionality to high phenylalanine have been suggested: 1) inter-individual variability at the level of blood-brain barrier decreasing the transport of phenylalanine to the brain (such as rs113883650 variant in the *LAT1* (*SLC7A5*) gene) and 2) escape mechanism in one of the metabolic pathways [4], [31]. We propose a third hypothetical mechanism which takes into account the complexity of dendritic branching and the number of synaptic connections for brain resilience, further suggesting that these three mechanisms do not exclude each other. Therefore, variants in *SHANK* genes together with the absence of rs113883650 variant in the *LAT1* (*SLC7A5*) in our patients may work together to bring protection from the intellectual disability.

If SHANK proteins bearing the identified variants prove protective modifier effect on cognitive development of PKU patients, their discovery could have further implications. It has been envisioned that the putative protector could be a target for therapeutic intervention in the treatment of PKU [47]. A novel role for glutamatergic synapse in the pathogenesis of PKU cognitive dysfunction could become the aim for the repurposment of appropriate drugs already in use in clinical practice, such as glutamatergic modulators, which could alleviate neurobehavioral disturbances of PKU patients. Repurposing drugs for new indications, such as to treat rare diseases, is a time-saving and cost-efficient method resulting in higher success rates, compared with the development of novel orphan drugs [48]. Consequently, this novel treatment may help in achieving optimal cognitive and psychomotor development of PKU patients, along with regular PKU diet, as well as contribute to better life quality for adult patients who discontinued the diet. Furthermore, this discovery opens the door for the possibility of development of novel drugs based on genotype.

There have been several reports pointing out that subjects escaping intellectual disability might very well be more common than we think, implying that these persons are being over-treated or treated in a suboptimal way [4], [31], [45], [49]. Therefore, discovery of genetic modifiers would be a step forward towards personalized treatment of PKU patients. Identification of genetic factors which could have protective modifier effect on cognitive dysfunction of PKU patients will therefore have impact not only for clinical practice through the implications of novel strategies for individualized treatment of PKU pateints, but it will also contribute to solving the great puzzle – revealing the mechanism by which phenylalanine damages the brain.

The following are the supplementary data related to this article.

Supplementary Table 1 Variants identified in genes related to central nervous system functioning in the cohort of untreated PKU patients without intellectual disability. Supplementary Table 1

## Authors individual contributions to the paper

Kristel Klaassen: Formal analysis, Investigation, Data Curation, Writing - original draft/ review & editing.

Maja Djordjevic: Investigation, Resources.

Anita Skakic: Investigation, Visualization.

Bozica Kecman; Investigation, Resources.

Radoje Drmanac: Methodology, Resources.

Sonja Pavlovic: Funding acquisition, Writing - review & editing.

Maja Stojiljkovic: Conceptualization, Project administration, Supervision, Writing - review & editing.

## Declaration of Competing Interest

none.
