# Supplementary material for: Untreated PKU patients without intellectual disability: SHANK gene family as a candidate modifier
Source: Mol Genet Metab Rep. 2021 Nov 19;29:100822. doi: 10.1016/j.ymgmr.2021.100822 (PMC8639809; doi:10.1016/j.ymgmr.2021.100822)
Supplement: Supplementary Table 1 — Variants identified in genes related to central nervous system functioning in the cohort of untreated PKU patients without intellectual disability. [file mmc1.docx]

Supplementary Table 1: Variants identified in genes related to central nervous system functioning in the cohort of untreated PKU patients without intellectual disability

| **Gene** | **Reference sequence** | **Genomic coordinates at hg19** | **Genetic variant** | | **Variant type** | **GnomAD frequency** | **Patient** |
| --- | --- | --- | --- | --- | --- | --- | --- |
|  |  |  | **Nucleotide change** | **Amino acid change** |  |  |  |
| AHNAK* | NM_001620.3 | chr11:62297448 T/G | c.4441A>C | p.Lys1481Gln | missense | 0.00000398 | P3 |
|  |  | chr11:62291328 A/C | c.10561G>T | p.Gly3521Cys | missense | 0.00000398 | P4 |
| AHNAK2 | NM_138420.4 | chr14:105419347 C/T | c.2441G>A | p.Arg814Gln | missense | 0.00137 | P2 |
| ANK3 | NM_020987.5 | chr10:61802453 G/A | c.13130C>T | p.Ser4377Leu | missense | 0.000115 | P2 |
| ANO10 | NM_018075.5 | chr3:43621938 C/G | c.499G>C | p.Val167Leu | missense | 0.0000279 | P1 |
| ATP8B3 | NM_138813.4 | chr19:1788909 C/T | c.3056G>A | p.Gly1019Asp | missense | 0.00304 | P3 |
|  |  | chr19:1805954 C/T | c.754G>A | p.Asp252Asn | missense | 0.0000242 | P3 |
| C10orf112 (MALRD1) | NM_001142308.3 | chr10:19417213 A/G | c.1007A>G | p.Tyr336Cys | missense | Variant not found in gnomAD genomes | P1 |
| C3orf58 (DIPK2A) | NM_001134470.2 | chr3:143692390 ACG/GGA | c.16_17delGGinsAC | p.Gly6Thr | missense | Variant not found in gnomAD genomes | P1 |
| C6orf130 (OARD1) | NM_145063.3 | chr6:41038990 A/G | c.65T>C | p.Phe22Ser | missense | 0.0000239 | P1 |
| CACNA1H | NM_021098.3 | chr16:1255223 G/A | c.2561G>A | p.Arg854Gln | missense | 0.000254 | P1 |
| CADPS2 | NM_001009571.3 | chr7:122047656 G/A | c.2666C>T | p.Pro889Leu | missense | 0.0000204 | P1 |
|  |  | chr7:122303362 G/A | c.715C>T | p.Leu239Phe | missense | Variant not found in gnomAD genomes | P1 |
| CDHR2* | NM_017675.5 | chr5:176017436 A/-* | c.3365del* | p.Asn1122MetfsTer6* | frameshift | Variant not found in gnomAD genomes | P1, P2 |
|  |  | chr5:176017443 A/C | c.3371A>C | p.Gln1124Pro | missense | Variant not found in gnomAD genomes | P1 |
|  |  | chr5:176004401 G/A | c.1196G>A | p.Gly399Asp | missense | 0.000141 | P2 |
| CES1 | NM_001025195.2 | chr16:55846933 A/C | c.968T>G | p.Val323Gly | missense | Variant not found in gnomAD genomes | P3 |
| CLDN23* | NM_194284.3 | chr8:8560727 C/G | c.819C>G | p.Asp273Glu | missense | 0.0000121 | P3 |
|  |  | chr8:8560630 C/G | c.722C>G | p.Pro241Arg | missense | 0.0000733 | P4 |
| CLDN8 | NM_199328.3 | chr21:31587790 T/C | c.454A>G | p.Ile152Val | missense | 0.0000438 | P2 |
| CNTNAP2 | NM_014141.6 | chr7:145813994 G/C | c.26G>C | p.Cys9Ser | missense | Variant not found in gnomAD genomes | P2 |
| CTBP2 | NM_022802.3 | chr10:126715159 -/ GCCGCAGGCTGGGGCTGCAGG | c.1173_1174insGCAGCCCCAGCCTGCGGCCCT | p.Pro391_Leu392insAlaAlaProAlaCysGlyPro | insertion | Variant not found in gnomAD genomes | P3 |
| DBH | NM_000787.4 | chr9:136508691 G/A | c.901G>A | p.Ala301Thr | missense | 0.000475 | P1 |
| DCDC2B | NM_001099434.1 | chr1:32674742 -/G* | c.47_48insG* | p.Asn16LysfsTer68* | frameshift | Variant not found in gnomAD genomes | P3, P4 |
| DNAH14 | NM_001367479.1 | chr1:225519206 C/A | c.9791C>A | p.Ala3264Glu | missense | Variant not found in gnomAD genomes | P4 |
| DOPEY2 (DOP1B) | NM_00513328.3 | chr21:37605161 G/A | c.2410G>A | p.Val804Met | missense | Variant not found in gnomAD genomes | P2 |
| EFHD1 | NM_025202.4 | chr2: 233546391 G/A | c.682G>A | p.Ala228Thr | missense | 0.00000398 | P2 |
| HTR2C | NM_000868.3 | chrX:114141848 A/T | c.1247A>T | p.Tyr416Phe | missense | Variant not found in gnomAD genomes | P1 |
| KCNV2 | NM_133497.4 | chr9:2718992 CGC/GGG | c.1253_1254delTCinsGG | p.Ile418Arg | missense | Variant not found in gnomAD genomes | P2 |
| LYPD2 | NM_205545.3 | chr8:143831772 C/T | c.307G>A | p.Gly103Arg | missense | 0.0000178 | P1 |
| MARVELD3 | NM_001017967.4 | chr16:71674877 C/G | c.1180C>G | p.Gln394Glu | missense | 0.0000279 | P2 |
| MED25 | NM_030973.4 | chr19:50339528 AG/CT | c.2012A>T | p.Gln671Leu | missense | Variant not found in gnomAD genomes | P1 |
| MFSD6L | NM_152599.4 | chr17:8701169 ATGTAGCTTTG/TCCT | c.1260_1270delinsAGGA | p.Phe420LeufsTer17 | frameshift | Variant not found in gnomAD genomes | P2 |
| NAPRT1 | NM_145201.6 | chr8:144659492 C/T | c.515G>A | p.Arg172Gln | missense | 0.000214 | P1 |
| NLGN2 | NM_020795.4 | chr17:7311648 CCC/GGG | c.74_76delCCCinsGGG | p.Ala25_Pro26delinsGlyAla | missense/in frame | Variant not found in gnomAD genomes | P1 |
|  |  | chr17:7320870 G/A | c.2260G>A | p.Gly754Arg | missense | 0.00498 | P2 |
| NPEPPS | NM_006310.4 | chr17:45662938 TG/CA* | c.723_724delGTinsCA* | p.Met241_Ser242delinsIleThr* | missense/in frame | Variant not found in gnomAD genomes | P2, P4 |
| NRP2 | NM_018534.4 | chr2:206641241 GCACT/TA | c.2712_2716delGCACTinsTA | p.His905_Cys906delinsSer | deletion | Variant not found in gnomAD genomes | P2 |
| OTOF | NM_194248.3 | chr2:26712596 C/A | c.910G>T | p.Asp304Tyr | missense | Variant not found in gnomAD genomes | P3 |
|  |  | chr2:26712599 AG/GA | c.906_907delCTinsTC | p.Phe303Leu | missense | Variant not found in gnomAD genomes | P3 |
| PCDHA4 | NM_018907.4 | chr5:140186979 GGGCCGCGGAGG/ AAGACACCGGGA* | c.207_218delinsAAGACACCGGGA* | p.Gly70_Gly73delinsArgHisArgAsp* | missense/in frame | Variant not found in gnomAD genomes | P3, P4 |
| PCLO | NM_033026.6 | chr7:82784833 -/ GCTGAGCTGGAGGCTTAGCAGGACCAAGAG | c.1124_1125insCTCTTGGTCCTGCTAAGCCTCCAGCTCAGC | p.Gln375_His5142delinsHisSerTrpSerCys | insertion | Variant not found in gnomAD genomes | P2 |
| PKP3 | NM_007183.4 | chr11:397151 G/A | c.650G>A | p.Arg217His | missense | 0.0000716 | P3 |
| PPP1R9B | NM_032595.5 | chr17:48226950 T/C* | c.923A>G* | p.Glu308Gly* | missense | Variant not found in gnomAD genomes | P1, P2 |
| PTPRT* | NM_133170.4 | chr20:41514552 G/C | c.109C>G | p.His37Asp | missense | 0.00035 | P2 |
|  |  | chr20:40827886 C/T | c.2542G>A | p.Gly848Arg | missense | 0.000181 | P3 |
| RIMBP2 | NM_015347.5 | chr12:130926738 ACTG/CCCT | c.1105_1108delCAGTinsAGGG | p.Gln369_Cys370delinsArgGly | missense/in frame | Variant not found in gnomAD genomes | P4 |
| RIMS1 | NM_014989.5 | chr6:73023308 GT/AC | c.4064_4065delGTinsAC | p.Ser1355Asn | missense | Variant not found in gnomAD genomes | P1 |
| **SHANK1** | **chr19:51170446 G/C** | **NM_016148.5** | **c.4771C>G** | **p.Pro1591Ala** | **missense** | **0.00001578** | **P2** |
| **SHANK2** | **chr11:70332231 G/GAATGGC** | **NM_012309.5** | **c.4166_4167insGCCATT** | **p.Pro1388_Phe1389insLeuPro** | **in-frame insertion** | **0.001525** | **P3** |
|  | **chr11:70858237 C/T** | **NM_012309.5** | **c.136G>A** | **p.Gly46Ser** | **missense** | **0.0003531** | **P3** |
|  | **chr11:70858321 C/T** | **NM_012309.5** | **c.52G>A** | **p.Asp18Asn** | **missense** | **0.00002133** | **P4** |
| **SHANK3** | **chr22:51169504 C/A** | **NM_001372044.2** | **c.5146C>A** | **p.Pro1716Thr** | **missense** | **0.003056** | **P2** |
| SLC15A5 | NM_001170798.1 | chr12:16347327 G/T | c.1544C>A | p.Ala515Glu | missense | 0.00404 | P1 |
| SLC24A1 | NM_004727.3 | chr15:65917415 CC/G | c.997_998delCCinsG | p.Pro333GlufsTer31 | frameshift | Variant not found in gnomAD genomes | P3 |
| SLC35F5 | NM_025181.5 | chr2:114476839 A/G | c.1388T>C | p.Phe463Ser | missense | 0.000591 | P2 |
| SLC45A4 | NM_001080431.2 | chr8:142228772 C/T | c.814G>A | p.Glu272Lys | missense | 0.0000838 | P2 |
| SLC4A9* | NM_001258428.2 | chr5:139740522 T/A | c.428T>A | p.Leu143Gln | missense | Variant not found in gnomAD genomes | P2 |
|  |  | chr5:139740525 A/G | c.431A>G | p.Asp144Gly | missense | Variant not found in gnomAD genomes | P2 |
|  |  | chr5:139747113 A/G | c.2194A>G | p.Met732Val | missense | 0.0000173 | P4 |
| SLC6A3 | NM_001044.5 | chr5:1414883 G/A | c.1079C>T | p.Ser360Phe | missense | Variant not found in gnomAD genomes | P4 |
|  |  | chr5:1414885 -/A | c.1078dup | p.Ser360PhefsTer78 | frameshift | Variant not found in gnomAD genomes | P4 |
| SLC9A5 | NM_004594.3 | chr16:67304922 G/A | c.2500G>A | p.Asp834Asn | missense | 0.0000442 | P2 |
| TH | NM_199292.3 | chr11:2192950 C/T | c.67G>A | p.Ala23Thr | missense | 0.000221 | P1 |
| TSPAN16 | NM_012466.4 | chr19:11437473 -/G* | c.690dup* | p.Trp231ValfsTer12* | frameshift | Variant not found in gnomAD genomes | P2, P4 |
| VPS13A** | NM_033305.3 | chr9:80018168 G/C | c.9206G>C | p.Arg3069Thr | missense | Variant not found in gnomAD genomes | P2 |
|  |  | chr9:79898309 C/G | c.3157C>G | p.Gln1053Glu | missense | 0.0000241 | P3 |
|  |  | chr9:79814879 A/G | c.119A>G | p.Asn40Ser | missense | Variant not found in gnomAD genomes | P4 |

*Variants or genes with different variants found in two patients.

**Gene with different variants found in three patients. Variants in SHANK gene family are given in bold.
